# Follow Your Heart: Trials and Tribulations of Sir Terence English and the First Successful Heart Transplant in the United Kingdom

**DOI:** 10.7759/cureus.42051

**Published:** 2023-07-17

**Authors:** Jay J Park, Sir Terence English

**Affiliations:** 1 Department of Neurosurgery, Stanford University School of Medicine, Stanford, USA; 2 Department of General Surgery, Norfolk and Norwich University Hospital, Norwich, GBR; 3 Centre for Global Health, Usher Institute, The University of Edinburgh, Edinburgh, GBR; 4 St. Catherine's College, University of Camridge, Cambridge, GBR

**Keywords:** follow your heart, transplant surgery, cadiothoracic surgery, multidisciplinary heart team, history of cardiac surgery, surgical innovation, open heart surgery, sir terence english, heart transplant, stories of discovery

## Abstract

The journey of heart transplantation in the United Kingdom (UK) has been marked by challenges and triumphs. Following a series of unsuccessful transplant attempts in 1968, a moratorium was imposed on the procedure. However, in 1979, Sir Terence English broke the national ban, by performing the UK’s first successful heart transplant at Papworth Hospital. This achievement opened doors for advancements in heart and lung transplantation and established the Papworth programme as a world leader in the field. Sir Terence’s legacy stands as a testament to the transformative power of determination, perseverance and teamwork in overcoming the moratorium, lack of financial support, difficult colleagues and the failure of his first transplant attempt. Through a comprehensive review of the literature, qualitative interviews and Sir Terence’s personal contributions, this article provides an account of his trials and tribulations, aiming to inspire and encourage physicians, surgeons and scientists in their pursuit of innovation in the field of medicine.

## Editorial

Introduction 

The United Kingdom (UK) has a rich history of medical innovations and discoveries, from the first successful vaccine to in-vitro fertilisation. British scientists and physicians have made monumental contributions to modern surgery, with pioneers, such as Joseph Lister, who invented antiseptic surgery. Over the years, British surgeons have also claimed multiple world's 'firsts', including intraocular lens implantation, total hip replacement and robotic surgery. However, heart transplantation was not amongst the UK's early surgical successes. In fact, it took 12 years after South African surgeon Christiaan Barnard's first successful heart transplant in 1967 for the UK to achieve its own breakthrough. This milestone was accomplished by Sir Terence English, whose journey we explore in this article.

In 1968, Donald Ross^1^, a highly accomplished cardiac surgeon with a remarkable medical career, embarked on the initial endeavours of performing heart transplants in the UK. Unfortunately, his efforts were marred by a series of failures, which ultimately resulted in a moratorium being imposed on any further attempts. Eleven years later, Sir Terence made history in July 1979 by performing the first successful heart transplant in the UK at Papworth Hospital (now known as Royal Papworth Hospital) in Cambridgeshire. Keith Castle, a 52-year-old man from London, was the fortunate patient who survived longer than five years after the transplant. Two more successful cases followed that same year, signalling a breakthrough stemming from the initial success of the procedure [1]. Achieving a 'first' in a complex and high-risk surgical procedure is fundamentally different from inventing a device or discovering a novel scientific concept. A successful heart transplant procedure has an impact that extends beyond the operating room and is contingent on its sustainability, generalisability and replicability. Although being a pioneer in a complex and high-risk surgical procedure is a significant achievement, it requires overcoming numerous hurdles to establish the procedure as a clinical practice. Sir Terence’s success came amid a scarcity of transplant centres worldwide, a ban on attempts by the Department of Health in the UK due to past failures and a limited number of successful cases globally, inciting ethical concerns.

That initial successful operation allowed Sir Terence to grow Papworth into one of the leading heart and lung transplant programmes in Europe and paved the way for many subsequent accomplishments, including the first heart-lung-liver transplantation in the world [2]. However, the journey to success included a complex concoction of technological, clinical, political and financial challenges that had to be overcome. Through a comprehensive review of the literature, qualitative interviews and Sir Terence’s personal contributions, our objective is to provide a detailed account of these hurdles and the perseverance required, highlighting the impact and legacy of his achievement. By sharing this story, we hope to inspire and encourage innovations in other medical fields, recognising that even the most complex and high-risk advances can be achieved with appropriate knowledge, determination and good teamwork.

Trials and tribulations 

Moratorium of 1973

The Moratorium of 1973^2^ on heart transplants in Britain laid foundations to significant challenges for Sir Terence and was the first major obstacle in his journey to develop a heart transplant programme in the UK. In 1973, Sir Terence, as a newly appointed consultant cardiothoracic surgeon, convened a formal meeting with his colleagues at Addenbrooke’s Hospital^3^ and Papworth Hospital to garner support for the UK's inaugural heart transplant programme, unaware that the British Chief Medical Officer (CMO) had already declared a moratorium on heart transplants earlier that year [3]. 

The moratorium was implemented during a period of global caution towards heart transplants [4] and ultimately following the attempts by Ross, who had also served as a mentor to Sir Terence [3,5]. Even during Ross’s initial surgeries, influential stakeholders at the National Heart Hospital had expressed opposition to heart transplantation [6]. As a result, Ross’s failures only fuelled the negative sentiment surrounding heart transplants, with the initial team becoming the target of a media frenzy and public scrutiny [7], including being mocked in the front cover of a satirical magazine Private Eye [1,8] and being criticised by colleagues across the country [6,8-10]. Public distrust was detrimental to transplant programmes as they are highly dependent on donor participation, and this was observed by the sharp decline in not only heart donation but also kidney donation rates [7]. In essence, the moratorium was not merely a surgical ban; it was rather an inevitable outcome of events that had significant social, financial and political implications [7]. Consequently, Sir Terence had to navigate a hostile bureaucratic landscape with minimal co-worker support while striving to secure funding for the UK's heart transplant programme [11].

During this period, Sir Terence was the only cardiothoracic surgeon who was actively pursuing a heart transplant programme in the UK despite the moratorium. His senior colleague at Papworth, Ben Milstein, provided moral support but refrained from personal involvement. This also meant that, unlike Ross, who had secure funding from the British Heart Foundation [1], Sir Terence had no prior funding whatsoever, whether for research or for developing a new transplant programme. Surprisingly, in 1977, the Department of Health seemed to moderate their initial position, and the Transplant Advisory Panel (TAP) chaired by the CMO devised strict criteria for centres looking to implement heart transplant programmes [3]. Sir Terence submitted a proposal titled 'Clinical Cardiac Transplantation in Cambridge'. However, the outcome was disappointing in that no central funding was to be invested in such a programme and no one-off operations were to be approved. 

**Figure 1:**
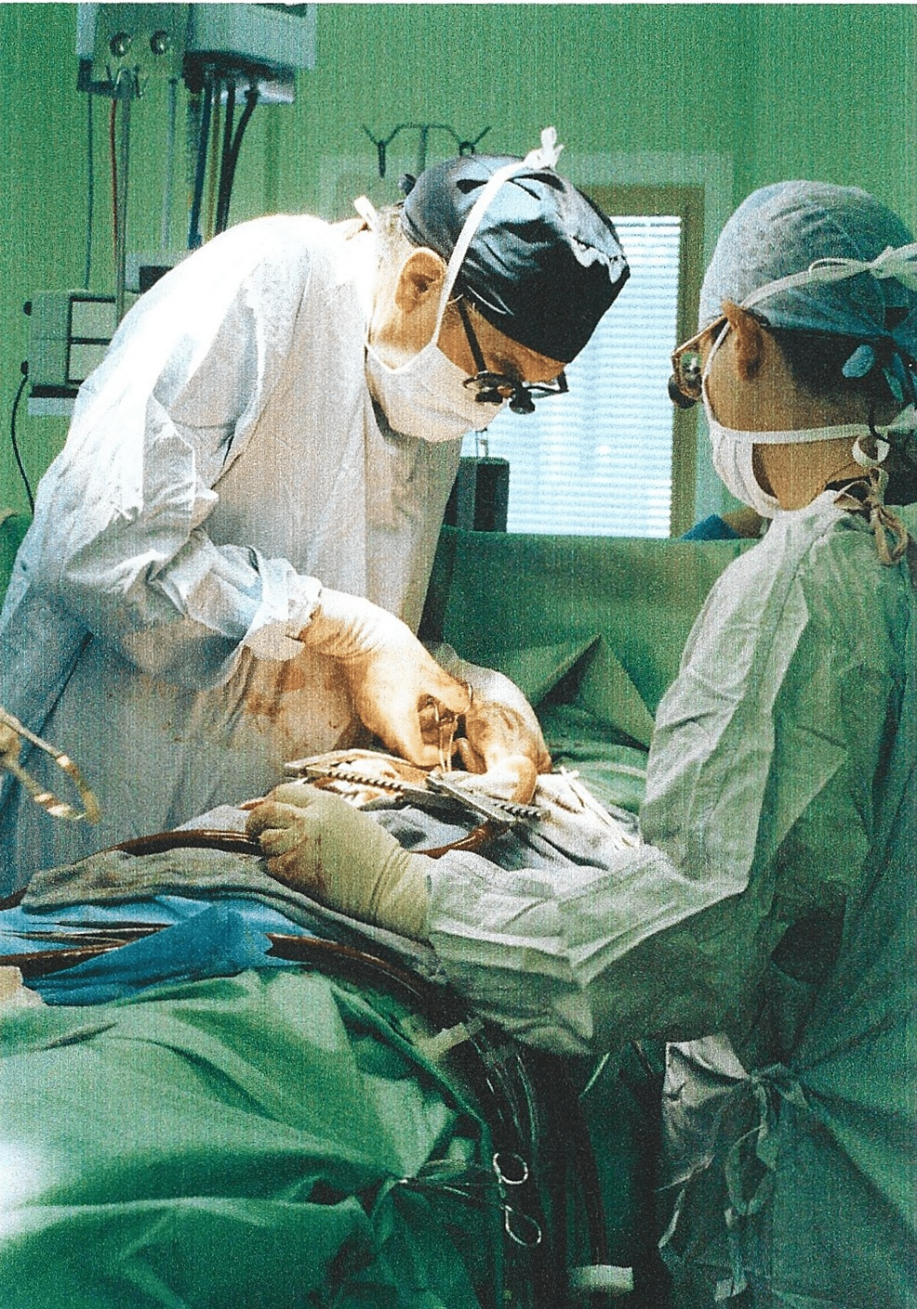
View of Sir Terence performing an open-heart operation.

Although the moratorium created an unfavourable environment for the initial launch of Sir Terence's efforts, he persevered. He took methodical steps to introduce experimentally a cardiac transplant programme in Cambridge. This involved training the necessary operating, intensive care and anaesthetic staff from both Addenbrooke’s and Papworth through a series of open-heart operations (Figure 1). He also continued to experiment with transplanting pig hearts, achieving increasing success. In October 1976, formal reports on the diagnosis of brain death were released, opening the possibility of extracting healthy hearts from brain-dead individuals. In 1977, Sir Terence received a grant for his two-year project investigating optimal methods for preserving donor hearts. Despite the disappointing outcome from TAP, he sought other alternatives. He met and discussed with Pauline Burnett, the Chairwoman of the Cambridge Area Health Authority (AHA), and her fellow officers who sympathetically supported him through the first two referrals by agreeing to use existing infrastructure at Papworth. This was the gateway to Sir Terence's first attempt at a heart transplant in 1979. Despite the discouraging circumstances stemming from the moratorium, Sir Terence's steadfast efforts prevailed, eventually bringing him closer to his vision that heart transplant was a necessity in the UK.

Financial Struggles 

Sir Terence aimed to establish a sustainable heart transplantation service within the British National Health Service (NHS) that could provide consistent care beyond just a single successful transplant. However, accomplishing this task within a public healthcare setting presented significant challenges as practicality and financial viability had to be considered. After Ross's attempts in 1969, the British Medical Association (BMA) Planning Unit questioned the practicality of heart transplants. They argued that this high-risk and high-cost procedure was not a solution to the general problem of degenerative cardiac disease but only for a handful of severely ill and desperate patients [7]. They also revealed a nuanced stance that 'occasional dramatised successes should not oblige the Health Service to try to meet the disproportionate demands of surgical enthusiasts for scarce medical, technical, and nursing resources that are implicit in a premature attempt to establish cardiac transplantation' [12]. Despite their intentions, the public image that Ross and his team set precedence in heart transplantation has discernibly prejudiced any financial decisions to support Sir Terence’s surgical ventures. Nevertheless, the unit also explicitly denied discouragement or prohibition of heart transplants. They clarified that their emphasis on the immediate practical benefits to the general population was not only due to the recent failures in heart transplants but also an inherent lack of national investment dedicated to research and the concerning level of overall expenditure on medicine in the UK compared to other developed countries [12]. This accurately depicts the climate of NHS budgeting that contributed to Sir Terence’s series of rejections from the Department of Health. 

Due to the lack of financial support from both the government and institutional sources, Sir Terence faced the daunting task of securing funding for his research as well as financing the transplants for his patients. Although Burnett and the AHA provided temporary support for the first two cases, this was far from sustainable funding to build a cardiac transplant program. Regrettably, Sir Terence’s initial patient did not survive long after the heart transplant. However, his fortunes changed when he achieved a successful second heart transplant in August 1979 for Keith Castle. This achievement enabled him to secure funding for the subsequent six patients through the National Heart Research Fund. Had it not been for his success with the second transplant, it is difficult to imagine how his journey would have progressed, given the reluctance of TAP to provide financial support during this critical period. Moreover, it was only after Sir Terence achieved two consecutive transplant successes that TAP acknowledged the heart transplant programme as a 'viable' proposal and escalated the decision to the Secretary of State for Health. Nevertheless, this had little impact on funding, and in January 1980, discussion around central NHS funding was again stalled. The CMO stated that the impact of a cardiac transplant programme on Papworth needed to be investigated by the Department of Community Medicine. Later, the department concluded that Papworth was capable of initiating a heart transplant programme. Meanwhile, Sir Terence continued to seek funding, whilst he garnered further successes with heart transplants. Following Burnett’s recommendation, he endeavoured to convince a local Cambridge millionaire, David Robinson, in supporting their work and Robinson ultimately agreed to provide the transplant programme for 1981 and 1982 with £300,000, along with additional coverage costs [3,13]. At the Parliament, the Secretary of State for Social Services recognised both the positive input from the Department of Community Medicine and the acquisition of Robinson funding in support of approving a cardiac transplant programme at Papworth.

Whilst clinically demonstrating the efficacy of cardiac transplantation supported by various funding sources, Sir Terence was finally granted a formal allocation of funding from the Department of Health and Social Security (DHSS). During a TAP meeting in October 1980, the British Transplantation Society criticised Sir Terence, alleging that Papworth was pursuing a programme that was neither planned nor funded by the DHSS. However, Sir Terence persisted and carried out more transplant cases, leading to the DHSS commissioning a three-year study into the costs and benefits of cardiac transplantation at Papworth Hospital and Harefield Hospital^4^. Although initially intended to explore costs only, Sir Terence and his colleagues stressed the importance of assessing the benefits of heart transplant on patients' duration and quality of life. The study ultimately concluded that there was 'little doubt of the effectiveness of the procedure in terms of improvement in the quality and quantity of life of transplanted patients' and convinced the DHSS to provide adequate resources to the heart transplant programmes at Papworth and Harefield in 1983 [3]. It was through Sir Terence's clinical and financial perseverance that the heart transplant programmes at Papworth and Harefield eventually secured Supra-Regional Funding. 

Difficult Colleagues

With the moratorium in place and no previous successful cases to reference, Sir Terence found himself without a mentor or guide amongst cardiothoracic surgeons in the UK. However, his determination was fuelled by witnessing the meticulous research and trials conducted by Norman Shumway^5^, also known as the 'Stanford protocol', during his week-long visit to Stanford University Hospital, which served as a key reference for him. Alongside the surgical challenges, Sir Terence also had to contend with significant obstacles in the form of lack of support and conflicts with colleagues, further impeding his efforts. These issues of dealing with difficult colleagues resonated deeply with Sir Terence, and he shares a personal narrative that vividly depicts the challenges he encountered.

'When the opportunity arose for me, as a newly appointed cardiothoracic surgeon, to be part of the transplant programme and research group led by renal surgeon, Professor Sir Roy Calne^6^, initially I was grateful - it seemed like a glimmer of hope. However, before long, my confidence in Calne’s judgments began to decline. One dramatic incident will illustrate this trend - early in our collaboration I returned from a meeting in Hong Kong to discover that Calne had planned to carry out a heart transplant personally in my absence but the Papworth team, including the anaesthetists, would not assist him! This episode was deeply disappointing as I had assessed that patient thoroughly and wrote in the notes that the patient was not a suitable candidate for a heart transplant, because he had gangrene of the feet and was too ill. Hugh Fleming, the one remaining cardiologist who was initially neutral about being involved in a cardiac transplant programme, had extensive agreement with my assessment. This incident completely alienated Fleming from continuing with the transplant work, leaving our team without a cardiologist [3,6]. My concerns about Calne’s clinical judgment increased when he insisted on the use of Cyclosporine, despite the drug’s largely unknown side effects. Subsequently, experience revealed that the use of Cyclosporine alone correlated with nephrotoxicity and a high rate of malignancy. These experiences reinforced my conviction that I must make my own decisions, even when it meant conflict with an established transplantation authority figure like Calne.

In addition, Calne did nothing to encourage the service departments at Addenbrooke’s Hospital to work with me. They fundamentally opposed a cardiac transplant programme being added to the burden of the established kidney and liver transplant programmes at their site. As a result, I determined that a separate heart transplant programme should be established at Papworth Hospital, led by experienced cardiac surgeons working in collaboration with skilled anaesthetists and theatre staff who had extensive expertise in treating cardiac patients. Throughout the initial trials Calne refused any collaboration if transplants were performed at Papworth. It is not an exaggeration to say that our success was possible because Calne was out of town for the two initial trials of heart transplants! Although he declined to participate in the press conference after the first transplant, Calne was very quick in denouncing the procedure when the patient passed away, asserting that I had set back the British cardiac transplant programme by five years. Calne further undermined our endeavours by formally raising the issue of separate cardiac facilities during the Transplant Advisory Panel (TAP) meeting, where he declared Cambridge to be ‘unsuitable’ for developing a cardiac transplant programme!

As well as requiring extensive specialised operating theatre staff, cardiologists were essential in the cardiac transplant team. However, I had much difficulty in securing support from the local cardiologists. After the very first meeting regarding the transplant programme two cardiologists from Papworth refused to be involved. One was Dr. Fleming and the other was Dr. David Evans, who rejected '…the clinical significance, moral and religious justification of the procedure'. Evans never moderated his criticism of heart transplantation and continued to speak publicly against it on ethical and religious grounds for the rest of his life.

We had brief periods of cardiologist, support when Michael Petch joined the team in 1977. However, much to our disappointment, Petch’s involvement ended abruptly in 1980 under the influence of Evans. This was a difficult period when the lack of suitable heart donors or recipients rendered factors, such as surgical capability, adequate facilities, staffing, availability of funding and approval from the authority inconsequential. I found it deeply frustrating that Calne persisted in failing to provide support or assistance in the search for donors and he remained disengaged from the cardiologists who shared the responsibility for helping to select recipients. Calne’s primary concern seemed to be the potential impact on kidney donations if heart donations were also requested and he actively obstructed our attempts to obtain hearts. He even initiated a policy to prohibit kidney retrievals if consent was also given for heart donation. At this stage, I began to explore new channels for obtaining hearts, including reaching out to a neurosurgeon at Addenbrooke’s and various neurosurgical centres in London. However, none of these efforts led to a positive result.

At this stage, I was facing extensive discouragement from close colleagues at the hospital but my determination to carry on was supported by a select few individuals who recognised the value of my vision. I was particularly grateful for the fact that Norman Shumway took the time to write letters of encouragement urging the pursuit of heart transplantation in the UK. In 1975, an unexpected and very welcome event occurred when I was at a surgical meeting in the USA and had breakfast with a friend, Hywel Davies - an excellent cardiologist. He had trained in the UK and before emigrating had applied for the consultant position at Papworth which went to David Evans! When Hywel heard of our need for an experienced cardiologist to work with the transplant programme, he immediately offered to come and join our team! I was able to recruit him because by this time we had some independent funding.

I also had the backing of my senior registrar David Cooper who worked with me in research studies on transplantation in animals. Even Calne’s senior registrar, Paul McMaster, went out of his was to identify viable hearts for my initial cases. My colleague Ben Milstein also offered generous support by actively supporting the recruitment of additional cardiothoracic Surgeons as the transplant programme gained momentum. I tried to keep my focus on actionable steps and to remain single minded in the pursuit of our goals, undeterred by individuals who were obstructive.'

The First Failure

After all the efforts to overcome the moratorium and all the difficulties with colleagues and lack of resources, sadly Sir Terence's first heart transplant proved unsuccessful when patient Charles McHugh died 17 days after surgery. McHugh had been a 44-year-old male with advanced heart disease at Papworth. Sir Terence had meticulously planned to personally carry out both the donor and transplant operations, aiming to ensure the highest level of precision. Undoubtedly, he felt a great deal of apprehension regarding the outcome of his inaugural attempt, recognising the multitude of consequences it would entail [14]. However, while he was taking out the donor’s heart at Addenbrooke’s, he was notified by the anaesthetist at Papworth that McHugh's heart needed resuscitation and there was potential for brain damage due to cardiac arrest. After a brief but comprehensive discussion with the anaesthetist, Sir Terence proceeded with the heart transplant, which was initially successful. McHugh was transferred to the intensive care unit following the operation, and his organs functioned well. Unfortunately, he did indeed suffer from a brain injury that prevented him from being taken off invasive ventilation, ultimately leading to his fatal lung infection. Although the heart recovered well, this was yet another failed heart transplant in the UK, 10 years after the initial series of attempts. 

During a critical time when success was desperately needed, the failure of Sir Terence’s first heart transplant only served to exacerbate the challenges faced in establishing the programme in the UK, both politically and financially. As a result of this setback, Sir Terence faced harsh criticisms from the TAP, which concluded that the allocation of funds was not justified at the time. The report also highlighted the lack of consensus between Calne and Sir Terence as a source of frustration for the Department of Health and Health Authority [6]. 

Despite the outcome, Sir Terence and his team remained undeterred by the first failure, believing that success with heart transplants could be accomplished and the programme should continue at Papworth. Sir Terence conveyed his conviction to the TAP by sharing his plan to pursue a second case as soon as a donor became available. He also handled the publicity surrounding the first transplant with caution to avoid further opposition. Everyone involved in the heart transplant was devastated by the outcome, but Sir Terence and his team remained resolute in their belief that the programme should continue.

Discussion

Sir Terence’s unfaltering spirit, and his determination to 'stick with it'^7^, prevailed against the political, financial and social adversities and the early death of his first heart transplant patient. He attributes the success of his early transplant work at Papworth and implementation of a cardiac transplant programme to the development of an enthusiastic and committed team. This comprised not only cardiothoracic surgeons but anaesthetists, led by the very supportive Don Bethune; technicians for the heart and lung machine; nursing staff; physiotherapists; and even ward administrators, all dedicated to the wellbeing of the patients. Sir Terence feels strongly that his challenging beginnings, involving conflicts with Calne, cardiology colleagues and meagre resources, drove the team to stand more united and to treat every patient with utmost care. The success of cardiac transplantation in the UK was not only a surgical accomplishment but also the creation of a new culture emphasising teamwork, innovation and perseverance.

The journey towards Sir Terence's ground-breaking innovation began well before he had established himself as a highly esteemed and internationally recognised cardiothoracic surgeon. It started at the very beginning of his appointment as a consultant, right after his training. It was during this early period that he dared to embark on a path of innovation. His determination inspired him to pursue his dream, undeterred by any unfavourable circumstances. Although a daunting prospect, his aspiration to establish a heart and lung transplant programme in the UK gave him the courage to challenge the conventions and overcome countless obstacles.

**Figure 2:**
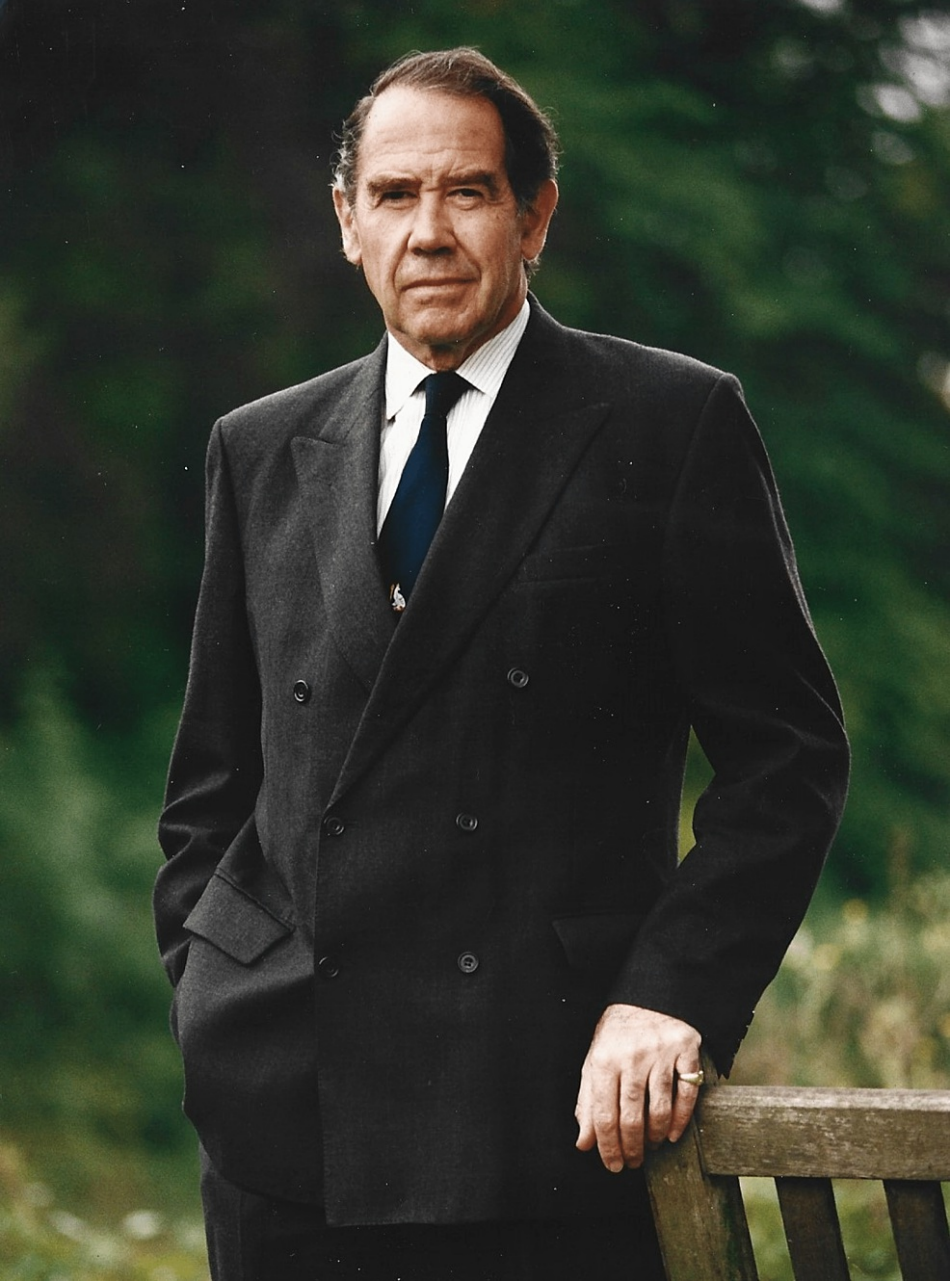
Photo of Sir Terence on his last working day at Papworth Hospital.

As Sir Terence embarked on his journey, something remarkable happened. By focusing on his true calling, instead of merely pursuing personal professional success, he found himself naturally compelled to acquire excellent technical surgical skills, become an innovative academic and evolve into a renowned figure in cardiothoracic surgery. His dedication ignited a fire within him, propelling him to reach unprecedented heights in his field. He recognised that genuine fulfilment and achievement are not measured solely by personal recognition or accolades. Instead, he understood that by focusing on the task ahead, success could eventually follow. In the fiercely competitive worlds of surgery and academia, where the pursuit of titles and prestige can often overshadow true purpose, a focus on his dreams served as a guiding light. His unwavering pursuit of excellence and commitment to teamwork set him apart from the crowd. By staying aligned with his true ambition, he not only established the UK's first successful heart and lung transplant programme but also inspired others to find their own paths to success. His story serves as a testament to the transformative power of following one’s heart, having lasting impact on the lives of countless patients and forever leaving an indelible mark on the field of cardiothoracic surgery in the UK and beyond. His legacy is that the Papworth programme is now the foremost centre for heart and lung transplantation in Europe (Figure 2).

Sir Terence highlights 'the importance of personal connections in role as a surgeon' (15). For example, he had valued the year during his training that he spent doing laboratory research with Professor John Kirklin in Birmingham, Alabama. Kirklin conducted a weekly team conference at which recent clinical cases were reviewed, with the emphasis on preventing recurrence of any problems. They remained in contact, and Kirklin encouraged Sir Terence during the establishment of Papworth Hospital's Cardiac Transplantation Programme. Later, in 1973, Terence visited Shumway at Stanford and became convinced that the UK should definitely have a heart transplant programme. He noted the strong team of bright residents in Shumway's unit and the related technical and laboratory research. They remained in contact long term and Shumway's backing was deeply appreciated. The moral support derived from those connections proved to be indispensable, empowering him to persist in the quest for innovation. Ultimately, despite the challenges that he faced whilst pioneering in cardiac transplantation, Sir Terence reflects that surgery is a very interesting career and urges young surgeons and innovators to be prepared to work very hard [15] and to 'follow your heart'^8^.

Footnotes

^1 ^Mr. Donald Nixon Ross, FRCS (1922-2014): South African-born British cardiac surgeon who pioneered open-heart procedures at the National Heart Hospital, London. He developed novel methods for hypothermia and the 'Ross procedure', which used the patient’s pulmonary valve to replace the aortic valve and a pulmonary homograft for the pulmonary valve replacement at the National Heart Hospital, UK [5]. Mr. Ross had 'superb hands' and was a brilliant colleague. During early days as a consultant, Sir Terence travelled to London during weekends to assist and learn from Ross, without payment [16].

^2^ Moratorium of 1973: Sir George Godber, the Chief Medical Officer of the British Department of Health, gathered experts who reported that heart transplantation was largely experimental. Worried about the cost implications, bad publicity, ethics and efficacy, he announced to the Regional Health Boards that no resources should be made available for heart transplants [1,3,17].

^3^ Addenbrooke’s Hospital: Tertiary teaching hospital based in Cambridgeshire, part of Cambridge University Hospitals NHS Trust. Its affiliated academic institution is University of Cambridge, along with the Royal Papworth Hospital. It hosted a range of transplant services at the time, including the kidney and liver.

^4^ Harefiled Hospital: Hospital (now under Guy’s and St. Thomas’ NHS Trust) just north of London, dedicated to heart and lung surgery, well known for the successful heart transplants by Sir Magdi Yacoub following Papworth.

^5^ Dr. Norman E. Shumway, MD, PhD (1923-2006): Cardiothoracic surgeon at Stanford University and was a pioneer in the early experimental work and research in heart transplantation. He dedicated his time towards refining the surgical technique and investigating ways to prevent cardiac transplant rejections. His unit’s numbers of transplanted hearts and success rate exceeded those in the rest of the world. Shumway, therefore, acquired the title of the 'father of heart transplantation' and was a valued mentor of Sir Terence [3,6,18].

^6^ Sir Roy Y. Calne, FRCP, FRCS, FRS (1930 - ): General surgeon and pioneer of kidney and liver transplantation based at Addenbrooke’s Hospital. He was the Professor of Surgery at Cambridge University from 1965 to 1998.

^7^ Personal advice given from Sir Terence when discussing my surgical and academic aspirations and innovative ideas. 

^8^ The title of this article was inspired by Sir Terence’s autobiography, 'Follow Your Star' [3].
